# Orthotopic Transplantation of Native and Cryopreserved Ovarian Tissue in Day-Old Geese

**DOI:** 10.3390/vetsci12020169

**Published:** 2025-02-13

**Authors:** Kitti Buda, Barbara Vegi, Istvan Lehoczky, Erika Meleg Edvine, Nora Palinkas-Bodzsar, Eva Kissne Varadi, Arpad Drobnyak, Judit Barna, Krisztina Liptoi

**Affiliations:** National Centre for Biodiversity and Gene Conservation, Institute for Farm Animal Gene Conservation, H-2100 Godollo, Hungary; vegi.barbara@nbgk.hu (B.V.); lehoczky.istvan@nbgk.hu (I.L.); meleg.erika@nbgk.hu (E.M.E.); palinkasbodzsar.nora@nbgk.hu (N.P.-B.); varadi.eva@nbgk.hu (E.K.V.); drobnyak.arpad@nbgk.hu (A.D.); barna.judit@nbgk.hu (J.B.)

**Keywords:** gonadal tissue transplantation, day-old goose, gene conservation, cryoconservation

## Abstract

As an alternative method for preserving female genetic material, the cryopreservation and orthotopic transplantation of day-old gonadal tissue have become well-developed techniques in a few poultry species. The aim of the present study was to apply these methods for the first time to domestic geese. Cryopreservation was accomplished using the previously developed vitrification technique. Native and frozen/thawed White Hungarian geese ovarian tissue was transplanted onto Grey Landes geese recipients. Both donors and recipients were less than 24 h old. The surgical protocol previously used in chickens needed to be modified regarding anaesthesia; ketamine, xylazine, and midazolam were administered partly intramuscularly and partly intravenously. After sexual maturation, the Grey Landes recipients were paired with White Hungarian ganders, and eggs were collected and hatched individually. The origin of the offspring was determined by phenotype- and microsatellite markers. Donor-derived offspring were obtained from native and frozen/thawed ovarian tissue transplantation, which had not been published earlier. The ratio of donor-derived progeny producing layers was 40% and 58% in native and frozen/thawed gonadal tissue transplantation, respectively. The highest rate of donor-originated progeny was 78.9%. Consequently, gonadal tissue transplantation appears to be a suitable method for preserving female genetic material in domestic geese; the technique is already implemented in Hungarian gene bank practice.

## Simple Summary

The aim of the present study was to develop a novel method for domestic geese that allows for the preservation of female genetic material in vitro and the 100% recovery of the entire genome in vivo as early as the first F1 offspring generation. At the day-old age, ovarian tissue were transplanted into recipients, first native and then after vitrification and thawing. After sexual maturity, the gonadal chimeric layers were paired with ganders of the donor genotype. The rate of layers producing donor-derived offspring was 40% and 58% after native and frozen/thawed gonadal tissue transplantation, respectively. The highest proportion of donor-derived offspring from a single recipient layer was 78.9%. Consequently, ovarian tissue vitrification followed by thawing and transplantation appears to be a suitable method for the long-term preservation of female genetic material in domestic geese and for the rapid and efficient recovery of the entire genome after fertilisation with donor sperm.

## 1. Introduction

Nowadays, the number of indigenous poultry breeds has declined significantly, with many of them reaching endangered status. Fourteen domestic chicken breeds have become extinct in the last 10 years according to the FAO report in 2015. This underscores the importance of poultry gene conservation, which primarily focuses on semen cryoconservation currently. However, in avian species, where males are the homogametic gender, freezing embryos or oocytes, which contain the W chromosome, is unfeasible due to their biophysical traits. Therefore, alternative methods are necessary to preserve female genetic material, such as gonadal tissue transplantation. This method involves the artificial insemination of gonadal chimaera hens with frozen/thawed semen from the donor breed, resulting in the complete restoration of the original genotype in the F1 generation. The technique of orthotopic transplantation of ovarian tissue at day-old age in domestic chickens was first published by Song and Silversides [1]. Since then, the cryopreservation and orthotopic transplantation of gonadal tissue at day-old age have become well-established techniques in various poultry species. Donor-derived progeny have been successfully obtained in Japanese quail and domestic chickens [2,3,4], while the vitrification and orthotopic transplantation of ovaries and testes in turkey has been performed by Hall [5]. While donor-derived progeny has been reported in waterfowl species, such as Muscovy ducks and Pekin ducks, using native ovarian tissue transplantation [6], there is a lack of similar publications regarding geese.

Our goal was to apply the previously developed cryopreservation and gonadal tissue transplantation techniques with necessary modifications in domestic geese.

## 2. Materials and Methods

The applied methods were approved by the Directorate of Food Safety and Animal Health of the Government Office of Pest Country, Hungary (PE/EA/729-8/2020). All chemical materials used were acquired from Merck Life Science Ltd. (Budapest, Hungary).

### 2.1. Animals

In this study, White Hungarian geese from the National Centre for Biodiversity and Gene Conservation (NBGK) gene bank stock and Grey Landes geese (Integral MB 09 breed) from Kisbér Lúd Ltd. (Godollo, Hungary) were used. White Hungarian is an indigenous Hungarian goose, which has white feathers, and an orange beak and legs; layers weigh 5–6 kg, while ganders weigh 6–8 kg. The yearly egg production is 30.

Grey Landes geese have grey cover feathers with an orange beak and legs; layers weigh 6–6.4 kg and ganders 7–7.3 kg. The yearly egg production is 50.

In Hungary, there are two indigenous goose breeds: White Hungarian and Frizzled Hungarian. We decided to choose White Hungarian as the donor and Grey landes as the recipient, because plumage colour can add information about the origin of the offspring. As Grey Landes recipients were paired with White Hungarian ganders after sexual maturation, the origin of the offspring can be determined by phenotype markers in addition to the molecular marker analysis.

The orthotopic transplantation of native ovarian tissue was performed with the White Hungarian geese donor/Grey Landes geese recipient combination. Then, the grafting of frozen/thawed ovarian tissue was completed in the same donor/recipient combination. Both the donors and recipients were less than 24 h old. Before the surgery, only water was provided ad libitum for them.

### 2.2. Removal, Vitrification and Thawing of Donor Gonadal Tissue

The donor day-old White Hungarian geese were euthanized by cervical dislocation. After the removal and the disinfection of the abdominal area, a 2–3 cm incision was made to open the abdominal cavity. The gastrointestinal tract was removed to access the ovaries, which were then excised using fine forceps and iris scissors. Then, the ovaries were cut into 2–3 pieces and drawn into acupuncture needles [7], then immersed in two different vitrification solutions containing Dulbecco’s Phosphate-Buffered Saline (DPBS) + 20% Fetal Bovine Serum (FBS) + 7.5% Dimethyl Sulfoxyde (DMSO) + 7.5% ethylene glycol (EG) and DPBS + 20% FBS + 15% DMSO + 15% EG + 0.5 M sucrose at room temperature for 5–5 min. After that, the gonads were placed in liquid-nitrogen-containing cryovials, which were put into nitrogen tanks. Between the removal and vitrification process, the gonads are kept in a vitrification solution for a maximum of 30 min. Before transplantation, the needles with the tissues were treated in three different thawing solutions for 5–5 min at 38.5 °C.

The contents of the thawing solutions were DPBS + 20% FBS and sucrose in increasing concentrations [4]. For achieving better adhesion, tissues were inspected after thawing, before transplantation under a stereomicroscope, and cleansed from any extraneous tissue, e.g., serous membrane, kidney pieces, and adrenal gland pieces, if needed.

### 2.3. Transplantation of Native and Frozen/Thawed Gonadal Tissue into Recipient Day-Old Geese

For performing the operation, the protocol used earlier in chicken was slightly modified. In case of anaesthesia, the combination of ketamine (15 mg/kg), xylazine (3 mg/kg) and midazolam (2 mg/kg) was administered partly intramuscularly and partly intravenously, then, if necessary, an additional Isoflurane mask was applied. The operations were conducted in heated room on a heated operating table to avoid pulmonary oedema. After removing the feathers, and the disinfection of the abdominal area with 70% ethanol, a median laparotomy was performed. By pushing the gastrointestinal tract aside, the recipients’ own gonads can be accessed. The ovariectomy was performed in a caudo-cranial direction, using fine forceps and iris scissors. Removal of the donor organ was made the same way as presented in the “Vitrification of donor gonadal tissue” section; subsequently, the native donor ovaries were cut in 2–3 pieces and placed in Dulbecco’s Modified Eagle Medium (DMEM) on ice until grafting for a maximum of 30 min. The donor organ piece was placed as close as possible to the anatomical position of the recipient’s own ovary. The abdominal cavity was closed in two layers—the abdominal wall separately and the subcutis and cutis together using a polyglycolic absorbable surgical suture (Safil C 4/0).

For performing frozen/thawed ovarian tissue transplantation, the thawing of the donor gonads was carried out according to the previously described method. The thawed ovarian tissue pieces are kept in DPBS + 20% FBS and a sucrose-containing solution for a maximum of 30 min prior to grafting. The anaesthesia and surgical technique corresponded to the native ovarian tissue transplantation.

### 2.4. Postoperative Treatment, Housing of the Animals

After the surgical procedure, 0.06 mg of dexamethasone per goose was administered intramuscularly to prevent an acute immune reaction. As a long-term immunosuppressant, mycophenolate mofetil at a dose of 4 mg/kg was given per os for two weeks individually. The recipients were housed in groups on straw litter, feeding and watering were ad libitum, and the temperature and light were programmed according to breeding technology standards. With the aim of reducing stress in the operated geese, daily training from day-old age was conducted. The animals were trained with feed motivation to facilitate handling and stay still during measuring, administering vitamins and medications, and checking the individual markers.

### 2.5. Progeny Tests

After sexual maturation, the Grey Landes recipients were paired with White Hungarian ganders and the eggs were collected and marked daily individually for 5 months. All eggs were incubated according to general protocol. Since gonadal tissue transplantation occurs at day-old age, preliminary characterisation of the donor animals is not possible; therefore, microsatellite marker analysis was performed on the parents and their offspring that seemed to be phenotypically donor-derived. In the case of native ovarian tissue transplantation, and in the case of frozen/thawed ovarian tissue grafting, 76 and 234 animals, respectively, were characterised using microsatellite markers. Blood samples were collected in Na-citrate (1:1) from the medial metatarsal vein of each offspring considered to be donor-derived based on their phenotype markers, as well as from their parents. Samples were stored in −20 °C until DNA isolation. For DNA extraction, the traditional salting-out method [8] was modified for the poultry species [9]. The DNA concentration was determined by Nanodrop 2000 spectrophotometer (Thermo Fisher Scientific, Waltham, MA, USA), and the samples were equilibrated to concentrations of 5 ng/μL [4]. Based on previous research conducted at the National Centre for Biodiversity and Gene Conservation and the Institute for Farm Animal Conservation, 5 out of 20 microsatellite markers were selected as suitable for differentiating between populations of the indigenous Hungarian goose breeds. The microsatellite markers APH20 [10] ANS21, ANS25, SMO76, and ANS7 [11] were used in multiplex PCR protocol, which made it possible to exclude the recipient origin of the offspring. The genotyping was performed by capillary gel electrophoresis system using an automated DNA sequencer (GenomeLab™ GeXP Genetic Analysis System, Beckman Coulter, Inc., 4300 North Harbor Boulevard, Fullerton, CA 92834-3100, USA) according to the manufacturer’s instructions. Allele sizes were determined using an allele ladder of 400 bp length (400 bp size standard). The results were analysed using the GenomeLab Genetic Analysis System software (Beckman Coulter, Inc., 4300 North Harbor Boulevard, Fullerton, CA 92834-3100, USA).

### 2.6. Statistical Analysis

Chi square test was performed for comparing the ratio of donor-derived progeny from native and frozen/thawed ovarian tissues.

## 3. Results

A progeny test from the transplantation of native and frozen/thawed ovarian tissue was performed on the White Hungarian donor/Grey Landes recipient combination.

**Figure 1:**
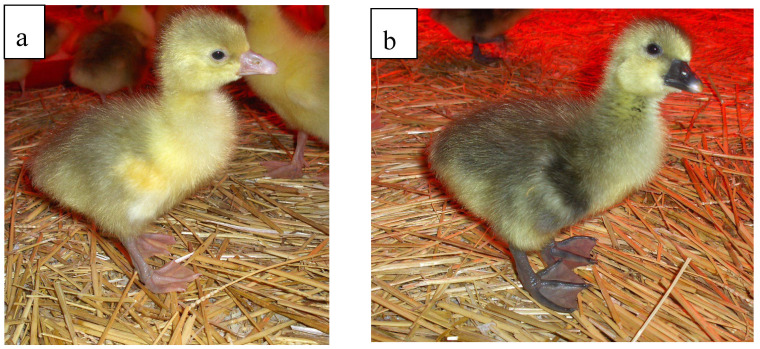
The donor-derived offspring (White Hungarian) have a pink beak and legs (**a**); the recipient-derived offspring (Grey landes) have a dark grey beak and legs at day-old age (**b**). In both cases, the day-old feathers are grey-yellow, while the adult colour is white for the donor-derived, and grey for the recipient-derived animals. In addition to the phenotype markers, the origin of the offspring was determined by microsatellite markers as well.

**Figure 2:**
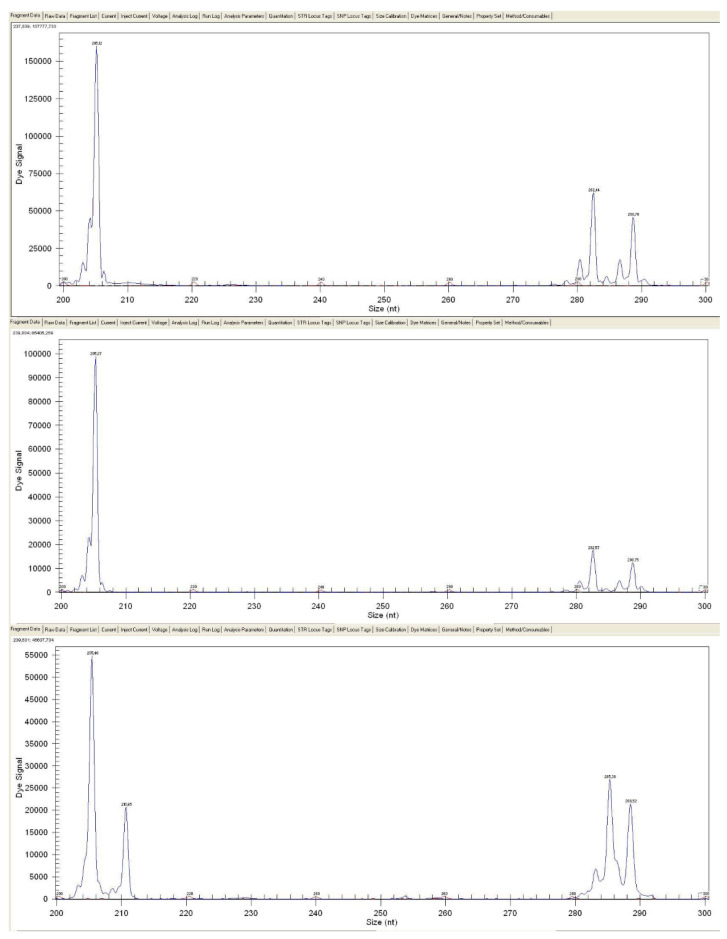
Fragment analysis performed with the GenomeLab Genetic Analysis System software using two microsatellite markers in a recipient female and a male goose, and their genetically proven donor-derived offspring. Upper part of the image: allele sizes detected in the recipient layer, ANS21: 205/205 basepair (bp) homozygote, ANS25: 282/288 bp heterozygote. Middle part of the image: allele sizes detected in the gander, ANS21: 205/205 bp homozygote, ANS25: 282/288 bp heterozygote. Bottom part of the image: allele sizes detected in offspring of them, ANS21: 205/210 bp heterozygote, ANS25: 286/288 bp. heterozygote. For both markers, the offspring had alleles (ANS21: 210 bp, ANS25: 286 bp) that could not have originated from either parent because they did not carry them, so these alleles may not have come from the recipient layer.

According to our results, presented in Table 1, the ratio of fertile eggs was nearly the same in the native and frozen/thawed transplanted groups. According to this result, no significant difference was found between native and frozen/thawed transplantation (*p* ≤ 0.9). In the case of native ovarian tissue transplantation of White Hungarian donor/Grey Landes recipients, the proportion of layers producing donor-derived progeny was 40%, while in the case of frozen/thawed ovarian tissue transplantation, 58% of recipient layers produced donor-derived offspring, which showed no significant deviation (*p* ≤ 0.6). The average ratio of donor-derived progeny in native grafting was 57.75% (ranging from 50% to 67%), while in the case of frozen/thawed it was 19.7% (3.6–78.9%). In those cases, where phenotype markers suggested the donor-derived origin of the offspring (Figure 1), microsatellite markers also excluded the recipient origin (Figure 2); 17 samples from 76 for the native ovarian tissue transplantation, and 54 from 234 for the frozen/thawed ovarian tissue transplantation, were confirmed as donor-derived. The colour determined at day-old age did not change after feathering out.

## 4. Discussion

Donor-derived offspring were successfully obtained from transplanted frozen/thawed ovaries, a finding which has not been published earlier. Nearly a quarter of the offspring were confirmed to be donor-derived, demonstrating sufficient efficiency for full genome recovery in domestic geese, for which no other method is currently known.

The adaptation of the ovarian tissue transplantation to domestic geese presented several differences compared to the technique developed for domestic chicken.

First, performing the ovariectomy was less complicated due to the larger size of the day-old geese and the gonad itself. Unlike in chicken, the removal of the yolk sac was unnecessary in geese due to its lower position. Since the yolk sac provides enough nutrients at the day-old age, food withdrawal did not cause any issues, at the same time supporting the gastrointestinal tract so as not to obscure vision during surgery. In chickens, extracting the ovaries using fine forceps and iris scissors without causing severe injury to the animals posed challenges [1,12], whereas in geese, the elongated ovary could be lifted and dissected by iris scissors more easily. However, achieving a full ovariectomy in geese was hindered by the proximity of the abdominal aorta and caudal vena cava, as damaging these vessels could lead to bleeding out in some cases. Since a fully functioning gonad can develop from every tiny piece of ovary, by performing partial ovariectomy, both donor- and recipient-derived progeny can be produced. This can result in wide individual variation in the production of the donor-derived progeny of recipient layers (3.6% to 78.9% in the case of frozen/thawed and 50% to 67% in the case of native transplantation). By improving the technique of the ovariectomy, the ratio of donor-derived progeny could be increased, but the lower success rate could also be enough to reconstruct the flock, since layers produce donor-derived offspring during their whole lifespan.

Secondly, the administration of anaesthesia in geese proved to be more challenging than in chickens due to the different metabolisms and the geese’s reactions to the anaesthetics. Presumably due to the accumulation of drugs in adipose tissue, increasing the doses caused cardiac arrest. Modifying the anaesthesia protocol by adding midazolam and administering partly intravenously and partly intramuscularly helped to achieve the proper level of anaesthesia [13].

In terms of postoperative care of the recipients, applying mycophenolate mofetil (CellCept) as an immunosuppressant was planned for 8 weeks [3,4,5]. However, in a study involving Japanese quail, mycophenolate mofetil has been applied only for 2 weeks and, comparing them with the non-immunosuppressed group, the ratio of donor-derived progeny was 69% higher in the immunosuppressed group [14]. In a Muscovy duck donor/ Pekin duck recipient combination, immunosuppressant administration also took place for 2 weeks [6]. Finally, in geese, we opted to administer the medication for 2 weeks as well, considering animal welfare and 3R protocol, since at an older age, drug administration caused increased stress of the recipients. Despite this, the adhesion rate of the donor gonads remained unaffected. Our results showed no significant difference in the ratio of donor-derived progeny between native and frozen/thawed ovarian tissue transplantation in geese, with rates of 22.4% and 23%, respectively. Although the origin of the progeny can be determined by phenotype markers, molecular marker analysis could provide a more certain result.

In previous studies, it was reported that 33% of Japanese quail recipients produced donor-derived progeny through the grafting cryopreserved ovarian tissue at day-old age [12]. In the case of native gonadal tissue grafting in a Muscovy duck donor/Pekin duck recipient combination, 25% of the recipients produced donor-derived progeny in 100% of cases [6]. While in our investigations, the highest ratio of donor-derived progeny producing layers was 58%.

## 5. Conclusions

In conclusion, gonadal tissue transplantation appears to be suitable method for preserving female genetic material in geese. The method can be performed under simple circumstances, and the resulting gonadal chimaera layers can produce donor-derived progeny throughout their lifespan. Moreover, the technique is being implemented in the Hungarian gene bank practice, as goose gonadal tissues are already stored in the gene bank.

## Figures and Tables

**Table 1:** Results of native and cryopreserved ovarian tissue transplantation in White Hungarian donor/Grey Landes recipient combination.

	Number of Recipient Geese	Ratio of Fertile Egg-Producing Layers	All Produced Eggs	Ratio of Donor-Derived Progeny According to Microsatellite Marker Analysis Among All Progeny
Transplantation of native ovarian tissue	10	80%	283	22.4%
Transplantation of cryopreserved ovarian tissue	17	88%	679	23%

## Data Availability

No new data were created or analyzed in this study. Data sharing is not applicable to this article.
